# Pericapillary Edema Assessment by Means of the Nailfold Capillaroscopy and Laser Scanning Microscopy

**DOI:** 10.3390/diagnostics10121107

**Published:** 2020-12-18

**Authors:** Boris P. Yakimov, Yury I. Gurfinkel, Denis A. Davydov, Anastasia S. Allenova, Gleb S. Budylin, Vladimir Yu. Vasiliev, Vera Yu. Soldatova, Armais A. Kamalov, Simon T. Matskeplishvili, Alexander V. Priezzhev, Evgeny A. Shirshin

**Affiliations:** 1 Faculty of Physics, Lomonosov Moscow State University, Leninskie Gory 1/2, 119991 Moscow, Russia; bp.jakimov@physics.msu.ru (B.P.Y.); da.davydov@physics.msu.ru (D.A.D.); avp2@physics.msu.ru (A.V.P.); 2 Institute for Regenerative Medicine, Sechenov First Moscow State Medical University, Trubetskaya 8-2, 119991 Moscow, Russia; 3 Medical Research and Education Center, M.V. Lomonosov Moscow State University, Lomonosovsky Prospect 27/10, 119991 Moscow, Russia; yugurf@yandex.ru (Y.I.G.); gleb.budylin@gmail.com (G.S.B.); armais.kamalov@rambler.ru (A.A.K.); simonmats@yahoo.com (S.T.M.); 4 Division of Immune-Mediated Skin Diseases, Sechenov First Moscow State Medical University, Trubetskaya 8-2, 119991 Moscow, Russia; erika-mma@yandex.ru; 5 Institute of Spectroscopy of the Russian Academy of Sciences, Fizicheskaya Street, 5, Troitsk, 108840 Moscow, Russia; 6 A.I. Yevdokimov Moscow State University of Medicine and Dentistry, Delegatskaya Street, 20, 127473 Moscow, Russia; prof.vl.vasilev@mail.ru (V.Y.V.); vera.soldatova.73@mail.ru (V.Y.S.)

**Keywords:** edema, nailfold capillaroscopy, perivascular area, laser scanning microscopy, papillary dermis, fluid accumulation, hyporefractivity, image sharpness assessment

## Abstract

Edema, i.e., fluid accumulation in the interstitial space, accompanies numerous pathological states of the human organism, including heart failure (HF), inflammatory response, and lymphedema. Nevertheless, techniques for quantitative assessment of the edema’s severity and dynamics are absent in clinical practice, and the analysis is mainly limited to physical examination. This fact stimulates the development of novel methods for fast and reliable diagnostics of fluid retention in tissues. In this work, we focused on the possibilities of two microscopic techniques, nailfold video capillaroscopy (NVC) and confocal laser scanning microscopy (CLSM), in the assessment of the short-term and long-term cutaneous edema. We showed that for the patients with HF, morphological parameters obtained by NVC—namely, the apical diameter of capillaries and the size of the perivascular zone—indicate long-term edema. On the other hand, for healthy volunteers, the application of two models of short-term edema, venous occlusion, and histamine treatment of the skin, did not reveal notable changes in the capillary parameters. However, a significant reduction of the NVC image sharpness was observed in this case, which was suggested to be due to water accumulation in the epidermis. To verify these findings, we made use of CLSM, which provides the skin structure with cellular resolution. It was observed that for the histamine-treated skin, the areas of the dermal papillae become hyporefractive, leading to the loss of contrast and the lower visibility of capillaries. Similar effect was observed for patients undergoing infusion therapy. Collectively, our results reveal the parameters can be used for pericapillary edema assessment using the NVC and CLSM, and paves the way for their application in a clinical set-up.

## 1. Introduction

Edema, i.e., fluid accumulation in the interstitial space, accompanies a number of pathological states of the human organism and has a different etiology. For instance, patients with heart failure (HF) often experience edematous syndrome [1], which results from both the activation of a series of humoral and neurohumoral mechanisms that promote sodium and water reabsorption by the kidneys, as well as from increased venous capillary pressure and decreased plasma oncotic pressure [2]. Other examples of pathophysiological processes leading to edema are lymphatic dysfunction (lymphedema) [3] and inflammation [4].

Because of the clinical need for edema quantification, the techniques to solve this task are in high demand. For instance, physical examination (e.g., using the “pitting scale”) and serial weighing remain the most used approaches for assessing edematous syndrome in HF. The review of eight clinical methods for edema quantification can be found in [5]. At the same time, numerous approaches based on different physical principles are suggested in the literature, including MRI, X-Ray computed tomography, ultrasonography, etc., which are deep tissue imaging techniques. On the other hand, a large body of work in the assessment of cutaneous edema employs optical methods, which can provide for molecular contrast, high sensitivity of detection, and are, in general, simpler. These methods include optical coherence tomography [6], multispectral imaging [6], optoacoustic spectroscopy [7], diffuse reflectance spectroscopy [8], Raman spectroscopy [9], terahertz imaging [10], and multiphoton imaging [11]. 

The principles of edema assessment using optical techniques can be divided into two groups: (i) direct detection of water content using its specific spectral fingerprints—absorption (diffuse reflectance spectroscopy and multispectral imaging), or vibrational (Raman and terahertz spectroscopy) bands, and (ii) detection of edema-related morphological features (optical coherence and multiphoton tomography). Recently, we suggested using the optical microscopy-based technique, nailfold video capillaroscopy (NVC), to assess HF severity. In most studies, NVC is used to diagnose connective tissue diseases based on the parameters of the nailfold capillaries, namely, capillary density, capillary dimensions, capillary shape, and presence or absence of hemorrhages are characterized [12]. The terminology and the protocols for the description of the NVC findings are standardized in the consensus paper written by experts of the EULAR Study Group on Microcirculation in Rheumatic Diseases and the Scleroderma Clinical Trial Consortium Working Group on Capillaroscopy [12]. In [13], one more parameter, which can be extracted from the NVC images, namely, the perivascular zone (PZ) size, has been suggested as the marker of edema, and the morphological origin of the PZ was determined using two-photon tomography. It occurred that the PZ in the NVC images, which looks like a light area surrounding the capillaries, corresponds to the viable epidermis. Next, a strong correlation was observed between the PZ size and HF severity as described by the NYHA (New York Heart Association) classification [13]. This fact was supposed to be due to the increased fluid accumulation in the viable epidermis of patients with more severe HF, experiencing the edematous syndrome. However, this hypothesis still has to be confirmed, and one of the ways to do it is to perform NVC while directly changing the amount of interstitial fluid. 

In this work, we performed two types of experiments to reveal the markers of interstitial fluid accumulation in the NVC data: venous occlusion and histamine application to the skin. In the first case, we were inspired by the works of Kaznacheev and coauthors [14], who suggested using a venous occlusion-based test to assess capillary permeability by analyzing hematocrit before and after the occlusion. In the case of histamine, cutaneous edema caused by capillary vasodilation occurs, and this test is often used to calibrate the methods for water content assessment (see e.g., [6]). In addition, we made use of the confocal laser scanning microscopy (CLSM), which allows for the investigation of the skin structure with subcellular resolution. The experiment design thus included two parts: (i) induction of short-term edema under the NVC control and (ii) assessment of the morphological changes in the skin during these tests by CLSM to reveal the NVC and CLSM parameters, which can be used to quantify edema severity in a clinical set-up.

## 2. Materials and Methods

### 2.1. Patients and Healthy Volunteers

A detailed description of the cohort participated in the study of edema caused by HF is given in our previous work [13]—this dataset was reanalyzed in this paper to calculate additional parameters, e.g., the mean apical diameter. Briefly, 129 adults were enrolled in the study, including 50 healthy volunteers without symptoms of cardiovascular diseases and visible edema and 79 patients suffering from heart failure (HF) of different severity from I to III functional classes according to the New York Heart Association classification [1]. All HF patients underwent all clinical examinations prescribed to patients with HF in accordance with the European Society of Cardiology recommendations. 

Pilot studies using NVC were also on performed on patients undergoing infusion therapy. The NVC measurements were performed prior to and after 3 L infusion in the department of toxicology. The idea of this experiment was to assess and visualize the emergence of the short-term pericapillary edema, which was expected to occur at high infusion volume.

The study complies with the Declaration of Helsinki, and the study protocol was approved by the Ethics Committee of the Medical Research and Education Center of Lomonosov Moscow State University. Informed consent was obtained from all individuals.

### 2.2. Wide-Field Capillaroscopy Measurements

Nail bed capillaries were imaged using the custom-build video-capillaroscope Kapillaroscan-1 (AET, Moscow, Russia) equipped with a high-speed acquisition CCD camera TM-6740 GE (JAI, Tokyo, Japan), the light source with continuous spectrum in the 350–800 nm spectral range and the varifocal lens, providing 50×, 100× and 400× magnifications. Nailfold capillaries of the third and fourth fingers of the left hand were assessed under controlled conditions with subjects in the seated position and the left hand positioned at a heart level. All participants rested for 15 min before the examination. All measurements were made in a quiet temperature-controlled room (22–23.5 °C). A detailed description of the experimental set-up and capillaries examination protocol is given in [13].

### 2.3. Confocal Laser-Scanning Microscopy Measurements

Commercially available confocal laser-scanning microscopy (CLSM) system VivaScope 1500 (Lucid Inc., Rochester, NY, USA) suitable for in vivo applications with an excitation wavelength of 830 nm and reported spatial resolution of 1.25 μm and vertical resolution of 3–5 μm was used to study the skin parameters in the model experiments with induced short-term edema. The used set-up allows obtaining images of 500 × 500 μm^2^, while the device also provides the possibility of scanning several adjacent areas to obtain a large (mosaic) field of view from 1 × 1 up to 8 × 8 mm^2^.

### 2.4. Short-Term Edema Induction Protocols

#### 2.4.1. Histamine Application Protocol

Histamine aqueous solution (5 mg/mL) was topically applied on the skin of volunteers and was delivered through skin using a commercially available iontophoresis device to induce artificial local edema. Histamine applications were made in the regions near the nail bed of the fourth finger of the left hand for the assessment of the NVC parameters, and in case of edema examination using laser-scanning microscopy, histamine was applied on the inner side of the forearm to the region of ~1 × 1 cm^2^. After 5–10 min from histamine application, significant local edema was observed in the application region with erythema surrounding the edema region. The visible edema and tissue swelling disappeared 1–2 h after the application, and skin erythema disappeared 3–4 h after histamine application.

#### 2.4.2. Venous Occlusion Protocol

To evaluate the influence of edema that occurred under venous occlusion, as suggested in Kaznacheev et al. [14], we used a standard venous occlusion test with an inflated cuff to stop venous blood flow in the arm for a short period of time. To create a venous occlusion, the cuff was put on the subjects’ shoulder, and was then inflated up to 100 mm Hg for 5–7 min. The parameters of the forearm and the fingers of the hand to which occlusion was applied were assessed.

### 2.5. Image Processing Algorithms

#### 2.5.1. Capillaries’ Image Sharpness Assessment Procedure

**Figure 1:**
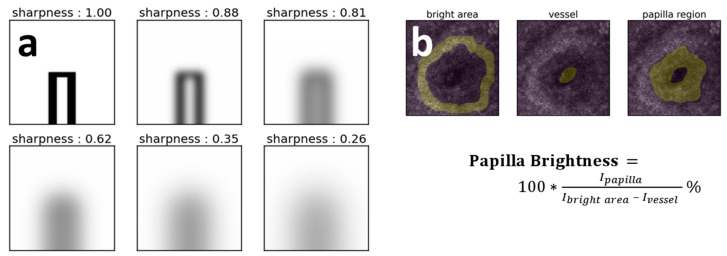
(**a**) Simulated images of microcapillaries blurred with a Gaussian filter with various σ parameter values and estimated sharpness. Gaussian filter values σ were equal to 0.5, 3.8, 7.2, 11, 14, and 17 (left to right, top to bottom). (**b**) Representative image of segmentation masks used to estimate papilla brightness in CLSM skin images.

To assess the edema effect on the NVC data, the sharpness of the capillaries images was evaluated. Since there are no standardized approaches in the field of image sharpness assessment yet, we used the following procedure to estimate image sharpness. As the first step, we applied a difference-of-Gaussians filter to the images where only first-row capillaries were located with filtration parameters σ_low_ = 0.5 and σ_high_ = 8. This type of filtration with selected parameters acted as a high-pass spatial frequency filter and allowed detecting only small objects with clearly visible borders, such as capillaries. As the second step, we calculated the average absolute value of the amplitude of the filtered image and considered it as a measure of the sharpness of an image. Figure 1a demonstrates the results of sharpness calculation on simulated images with a single model capillary blurred using Gaussian filtering with σ parameter varied from 0.5 to 17.

#### 2.5.2. Vessel Size Estimation in the CLSM Images

Since vessels in the CLSM images appeared as black areas surrounded by a bright white circular area, which corresponds to melanin located mainly near the dermal-epidermal junction, we used the following algorithm to estimate the vessel size. We first manually segmented the regions of interest corresponding to capillaries’ sections in the 4 × 4 mm^2^ CLSM images using the ROI manager in ImageJ software. Next, each segmented region was blurred with a Gaussian filter with σ = 15 to filter noise and irregularities in the images. After that, the smoothed image pixels with an intensity lower than 20% of the image’s maximum intensity were considered to the vessel. Vessel size was then estimated as the square root of the vessel region area.

#### 2.5.3. Papilla Brightness Estimation in the CLSM Images

In the 4 × 4 mm^2^, CLSM images, regions corresponded to the capillary sections were manually segmented using ImageJ software, such that each segmented area included a vessel, papilla, and bright circle corresponding to melanin located near the dermal-epidermal junction. In the segmented capillary images, dark regions corresponded to papillae, and bright regions corresponded to melanin. Papilla brightness was estimated as the ratio between the average intensity of the papilla region and the difference of average intensities of the bright epidermis area and dark vessel region (Figure 1b).

The use of the papilla brightness ratio should be standardized in at least two ways. First, one should avoid using the absolute value of intensity detected from papillae to estimate its brightness, as it depends on many parameters, including tissue absorption and scattering properties, scanning depth, laser scanning intensity, etc., and use papilla brightness relative to the intensity of some other objects instead. Second, one should be aware of the number of capillaries to count for the evaluation of the difference between papilla brightness for different subjects or before and after treatment and estimation of the statistical significance and the size of the effect. 

Here we estimated the brightness of each papilla as a ratio of intensity in the area of papillae and difference of intensities in the melanin-related bright “ring” located near the dermal-epidermal junction and capillary loophole using a semi-automatic segmentation routine. The obtained values were additionally multiplied by a factor of 100 to get a convenient range of values. The use of the intensity ratio instead of absolute values of the intensity scattered from papillae allows avoiding artifacts associated with local changes in contrast that can be observed from image to image.

It can be estimated that the number of 500 × 500 μm^2^ images required for measurement of the papillae brightness should be on the order of 10–12 for each investigated subject, in a case when a large number of capillaries is well observed. 

All data analysis was performed using a custom-made script based on the Python programming language with Matplotlib, NumPy, Pandas, Scikit-image, and SciPy libraries.

## 3. Results

### 3.1. Capillaroscopic Assessment of Patients with HF

As a first step, we analyzed whether the parameters provided by the NVC could be indicative of the long-term edema in patients with HF. For this, we made, used, and reanalyzed the NVC dataset first published in [13].

**Figure 2:**
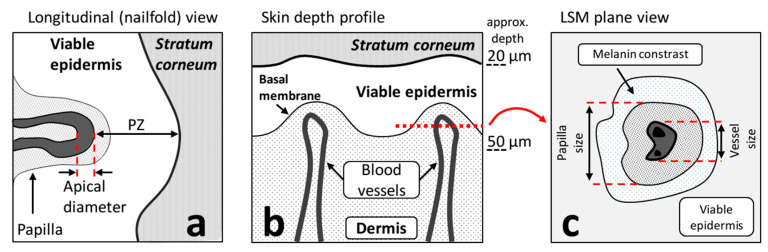
(**a**) Schematic of the nailfold cross-section, as imaged by NVC, and the extracted parameters. (**b**) Schematic of the skin structure. The red dotted line corresponds to the cross-section near the basal membrane. (**c**) Schematic of the typical CLSM image measured near the basal membrane, which includes the cross-section of the dermal papilla and a superficial capillary.

NVC provides for the image of the superficial capillaries of the nailfold, which are surrounded by the area referred to as the perivascular zone (PZ; Figure 2a).

**Figure 3:**
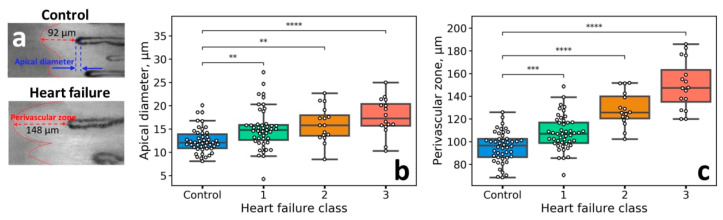
(**a**) Representative images of the nailfold capillaries for the control subject (upper panel) and patient with HF (NYHA class III, lower panel), adapted from [13]. The widths of the PZ are shown with a red dashed line. The dependence of the (**b**) apical diameter, and (**c**) PZ size on the NYHA HF class. The statistical significance was estimated using Kruskal-Wallis test with Bonferroni correction (* *p* < 0.05; ** *p* < 0.01; *** *p* < 0.001; **** *p* < 0.0001, ns—not significant).

It was previously shown that the border of PZ morphologically corresponds to the border between the viable epidermis and stratum corneum [13]. Moreover, the size of the PZ was found to be directly related to the HF severity as graded by the NYHA classification [13]. This fact was explained as the consequence of the viable epidermis swelling caused by fluid accumulation. In this work, we questioned whether other parameters, which are classically retrieved in capillaroscopy studies [12], are also indicative of the HF severity. Representative images obtained with NVC for the control subject and patient with HF are shown in Figure 3a. It was revealed that the apical diameter of capillaries exhibited a direct relationship to the NYHA HF class—the higher the HF grade, the larger the capillary diameters (Figure 3b). Although this parameter allowed for a poorer discrimination between the NYHA HF groups compared to the PZ size (Figure 3c), it can find its application due to the simplicity of the apical diameter estimation.

### 3.2. Short-Term Edema Models

As the next step to verify the relevance of the capillaroscopy parameters to assess the pericapillary edema, we employed two models. The relevance of the PZ size to fluid accumulation is based on the indirect evidence that includes the following observations: (i) the PZ sized correlates with HF severity, which itself is accompanied by the edematous syndrome, and (ii) that the PZ size corresponds to the size of the viable epidermis, which is susceptible to the fluid accumulation due to an increase of the intercellular space [13]. However, a direct evidence that the PZ size is governed by the water content in the viable epidermis was lacking. We would also like to emphasize that in the literature devoted to capillaroscopic investigations the presence of edema is sometimes described [15,16,17,18], however, to the best of our knowledge, there is no a specific parameter or precise description of how edema should look like in the capillaroscopic images. To address this question, we studied alterations of the NVC images following the external influences, which should result in increasing the water content in the perivascular space.

Firstly, we made use of the venous occlusion test. During venous occlusion, the amount of blood in vessels increases, and the extra liquid is extravasated through the vessels’ walls. This approach was used in the method of Kaznacheev et al. [14], which allows assessing the transcapillary diffusion. By comparing the hematocrit of the arterial and venous blood before and after venous occlusion, the amount of blood plasma that leaked out of the vessels can be calculated. Hence, we employed this setting to increase the amount of water in the perivascular area.

Secondly, the skin surface was treated with histamine, which induced capillary dilation and consequent appearance of the edema. This model has been multiply described in the literature [6].

To get an in-depth understanding of morphological changes, which underlay the possible (expected) alterations of the capillaroscopic images, we backed them with the CLSM measurements. CLSM provides for the depth-resolved skin structure with the subcellular resolution and allows for morphological assessment of structures observed with capillaroscopy.

### 3.3. Venous Occlusion

In the venous occlusion test, we did not observe any significant changes either of the nailfold capillary parameters or of the PZ size. We supposed that the amount of extra liquid, which accumulates in tissues from microcapillaries, is not large enough to induce swelling of the viable epidermis. However, notable changes were observed in the CLSM images following venous occlusion.

**Figure 4:**
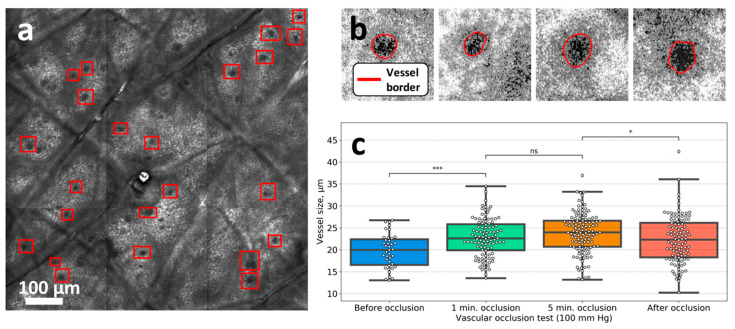
(**a**) CLSM image of the human forearm obtained at 50 mkm depth. The red rectangles correspond to the areas containing capillaries. (**b**) CLSM images of the dermal capillary area measured during venous occlusion. The area marked red corresponds to the capillary. (**c**) Changes in the dermal capillary sizes during venous occlusion are presented as the box plots. The dots in each box plot correspond to individual capillaries. The whiskers represent the 1.5 interquartile values range in the data. The statistical significance was estimated using Kruskal–Wallis test with Bonferroni correction (* *p* < 0.05; ** *p* < 0.01; *** *p* < 0.001; **** *p* < 0.0001, ns—not significant).

Typical CLSM image measured at ca. 50 mkm depth is shown in Figure 4a. In the CLSM images, the capillaries look like dark spots inside the grey areas, which correspond to the dermal papillae, which, in turn, are normally surrounded by the bright circular region corresponding to the melanocytes near the basal membrane [19]. Schematic representation of the dermal papilla cross-section as obtained with CLSM is presented in Figure 2c. To assess the changes in the parameters of the capillaries, we performed the following procedure. The areas of the CLSM images containing dark spots, which correspond to the capillaries, were marked with rectangles (Figure 4a)—a representative example of the capillary image is shown in Figure 4b. Thus, the area of capillaries can be obtained and quantified from the CLSM images [20], and in this work, we performed such quantification using the custom-made software (see Materials and Methods for details). We observed an increase in the median capillary size 1 min after venous occlusion by 10% (Figure 4c). This increase is in agreement with the literature data, for instance, using NVC, Mathura et al. [21] observed an increase in the mean capillary diameter from 11.2 (in rest) to 12.1 mkm (during venous occlusion). However, we did not observe a significant increase in the mean capillary diameter during venous occlusion using NVC, as well as of any other parameters such as the number of perfused capillaries, capillaries shape, or the size of the PZ. The latter fact, from the point of view of the considered hypothesis, suggests that there is no fluid accumulation in the papillary layer of the dermis in the viable epidermis large enough to be revealed by NVC. Hence, the venous occlusion can not be employed as the model of short-term edema to demonstrate the analytical capabilities of the NVC in the edema assessment.

### 3.4. Histamine Application

Histamine treatment is a well-known method to induce capillary dilation and short-term edema. This approach, indeed, resulted in a notable change of the CLSM and NVC images.

**Figure 5:**
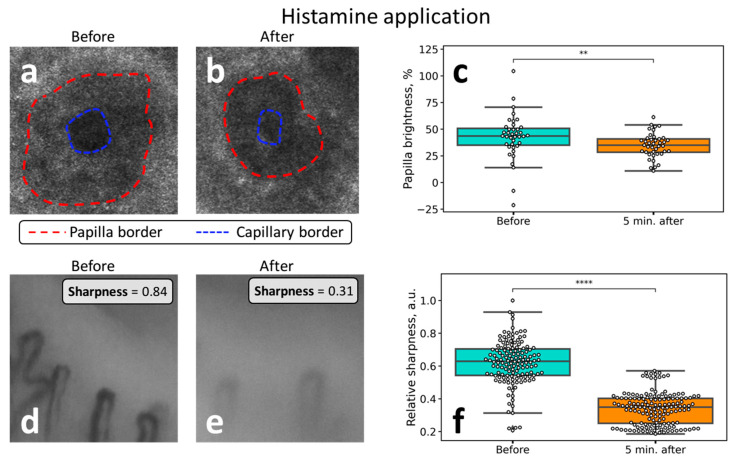
The images of the human forearm dermal papilla obtained with CLSM (**a**) before and (**b**) after histamine application to the skin. (**c**) The relative brightness of the dermal papillae in the CLSM images obtained before and after histamine application—the papillae become hyporefractive after treatment. The images of the human nailfold capillaries were obtained with the NVC (**d**) before and (**e**) after histamine application. (**f**) Changes of the relative sharpness (contrast) of the capillaries before and 5 min after histamine application. A significant decrease in visibility can be observed after treatment. The statistical significance was estimated using Kruskal–Wallis test with Bonferroni correction (* *p* < 0.05; ** *p* < 0.01; *** *p* < 0.001; **** *p* < 0.0001, ns—not significant).

The representative CLSM images of the dermal papilla before and after histamine application to the human forearm are shown in Figure 5a,b, respectively. It can be seen that after treatment with histamine, the area of the papilla becomes less contrasted—for instance, in Figure 5b (after treatment), it can be hardly separated from the area of the capillary (see the schematic in Figure 2c). Hence, the papillae become hyporefractive, i.e., light is scattered less efficiently compared to the initial state. This fact is quantified in Figure 5c, where the decrease in the brightness of the papillae is presented as the box plots.

Histamine application also had a pronounced effect on the NVC images. The most notable fact was the decrease in the image contrast (sharpness), which is demonstrated in Figure 5d–f. Hence, “cloudy” or “foggy” NVC images can be considered as a marker of cutaneous edema and fluid accumulation in the interstitial space and viable epidermis. The observed changes are summarized in Table 1.

**Figure 6:**
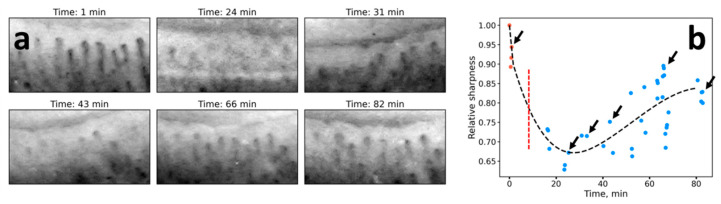
(**a**) The images of the human nailfold capillaries obtained with NVC before and after histamine application. Histamine was topically applied to the region of the nailfold. (**b**) The time course of the NVC image sharpness during histamine application. Each point represents the value of the estimated image sharpness. The dashed black line represents the fit of sharpness dependence on time using the least-square spline. The vertical dashed line represents the histamine application time; black arrows point to the values of capillaries images represented in panel (**a**).

We also assessed the time course of NVC image sharpness in the region of histamine-induced edema in the nailfold. Figure 6a demonstrates typical capillary images acquired at 100× magnification before and after histamine application to the nailfold region. The kinetics of decrease and increase of image sharpness is shown in Figure 6b. After the histamine application (vertical dashed line in Figure 6b), image sharpness rapidly decreased twofold, and then gradually reached the initial level.

**Figure 7:**
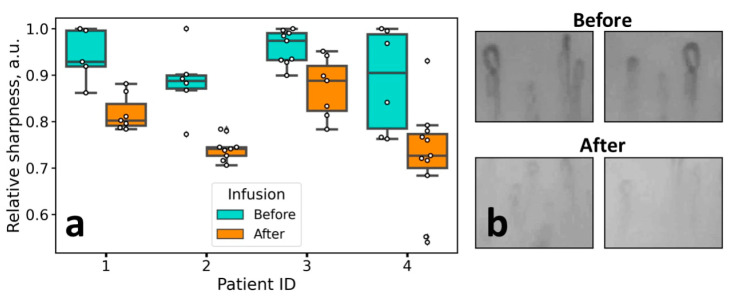
(**a**) Changes of the NVC image sharpness in patients before and after infusion therapy. Each dot corresponds to a single capillary. (**b**) Representative images of the capillaries before and after infusion therapy (after 3 L of infusion).

From the results, we came to the following conclusions: (1) long-term edema, e.g., in patients with HF, results in the increase of the PZ size, without visible alteration of the sharpness of the NVC images (Figure 3a), and, contrary to that, (2) short-term edema, e.g., caused by histamine-induced vasodilatation, does not increase the PZ size but rather decreases the sharpness of the NVC images (Figure 5 and Figure 6). While the later conclusion is important for quantitative interpretation of the NVC data when assessing edema, the diagnostic value of the image sharpness has to be further verified. To do this, we performed a preliminary study of patients undergoing infusion therapy. The idea of this experiment was to observe the short-term pericapillary edema, which could be caused by large infusion volumes (3L). The results of the experiment are shown in Figure 7: indeed, we observed a significant decrease in the NVC image sharpness after infusion, thus indicating the emergence of pronounced short-term edema. Hence, the image sharpness can be used as a diagnostic parameter to quantify the dynamics of pericapillary edema, e.g., in patients undergoing infusion therapy.

## 4. Discussion

The idea of using the PZ size, a non-conventional NVC parameter, occurred to be productive in the assessment of the HF severity [13]. In this work, the analysis of the dataset of the NVC images of patients with HF from [13] revealed that the apical diameter of capillaries also correlates with the HF severity as determined by the NYHA classification (Figure 3). The hypothesis, which initiated the current study, stated that the increase in the PZ size is caused by fluid retention in the interstitial space and viable epidermis, thus, we aimed at changing water content in the epidermis by altering either capillary blood pressure by venous occlusion or capillary permeability by histamine application. While the expected increase (~10%) was observed for the mean capillary diameter during venous occlusion, the parameters of the PZ remained constant. 

The most important results were obtained in the case of histamine application. It was observed that along with the edema emergence, the sharpness of the image decreased significantly, and this change was reversible. Hence, the sharpness of the NVC images is directly related to the water content in the epidermis—this fact is logical considering the fact that the light propagation, light penetration depth, and imaging contrast in tissues is mainly determined by light scattering, which, in turn, depends on the difference of the refractive index between the structures in the skin [22]. That is, the process of optical clearing, which allows improving the image contrast, is based on the idea to decrease the difference in the refractive index between the cells and extracellular space, e.g., by replacing water molecules with the clearing agent [23]. Notably, in a number of papers dealing with NVC, the “cloudy” appearance of the images is referred to as an indicator of edema [15,16,17,18]; for instance, in the recent paper on the COVID-19 microvascular involvement, pericapillary edema signs are described as a “foggy appearance around capillaries due to fluids build-up” [15]. Our results obtained with histamine confirm this interpretation, moreover, we suggest quantitative metrics to numerically characterize the degree of this foggy appearance (see Figure 1a and Figure 5d–f). The reversible change of the NVC image sharpness following histamine application is shown in Figure 6. These considerations are further supported by the CLSM data, where a decrease in the dermal papillae visibility (hyporefractivity) was observed after histamine application. Hence, we consider that the short-term edema models are not capable of altering the PZ size, i.e., the size of the viable epidermis, which is only modified during long-term edema, e.g., for the patient’s HF. The NVC image brightness, in turn, can be used as an indicator of pericapillary edema in a clinical set-up. This opportunity was tested in a pilot experiment on patients undergoing infusion therapy, which revealed a significant decrease in the image sharpness after infusion. Hence, although the NVC image sharpness does not allow for assessing the long-term edema as revealed by the example of patients with HF (Figure 3a), we consider that it can serve as a diagnostic parameter for quantification of the short-term edema, that was supported by the results of model (histamine application) and clinical (infusion) studies.

Summarizing our previous work [13], we have suggested the PZ size as an indicator of HF severity. In this study, we observed that this parameter was not indicative in the case of short-term edema in model experiments. At the same time, here we suggest another parameter, NVC image sharpness, which occurred to correlate with the edema emergence both in model experiments (Figure 5 and Figure 6), as well as in a clinical set-up (Figure 7, patients undergoing infusion therapy). Although the image sharpness seems to be of no diagnostic value in the case of HF (Figure 3a), it proved to be a promising tool for quantification of the short-term edema in dynamics.

## Figures and Tables

**Table 1:** Alterations of the NVC and CLSM parameters after venous occlusion and histamine application to the skin.

Application	NVC	CLSM
Venous occlusion	• no changes	• a 10–15% increase in the capillary diameter after occlusion
Histamine application	• a twofold decrease of the capillaries’ sharpness after histamine application	• a 20% decrease in the papillae brightness (hyporefractivity) following histamine application
